# Sprouty2 suppresses progression and correlates to favourable prognosis of intrahepatic cholangiocarcinoma via antagonizing FGFR2 signalling

**DOI:** 10.1111/jcmm.13833

**Published:** 2018-08-30

**Authors:** Yun‐Fei Xu, Hong‐Da Liu, Zeng‐Li Liu, Chang Pan, Xiao‐Qing Yang, Shang‐Lei Ning, Zong‐Li Zhang, Sen Guo, Jin‐Ming Yu

**Affiliations:** ^1^ Department of General Surgery Qilu Hospital of Shandong University Jinan China; ^2^ Department of Pharmacology & Chemical Biology University of Pittsburgh Pittsburgh Pennsylvania; ^3^ Department of Emergency Medicine and Chest Pain Center Qilu Hospital of Shandong University Jinan China; ^4^ Department of Pathology Qianfoshan Hospital of Shandong University Jinan China; ^5^ Department of Radiation Oncology Shandong Cancer Hospital Affiliated to Shandong University Jinan China

**Keywords:** FGFR2, intrahepatic cholangiocarcinoma, prognosis, sprouty, tyrosine phosphorylation

## Abstract

Fibroblast growth factor receptor 2 (FGFR2) was demonstrated to correlate to the progression and prognosis of intrahepatic cholangiocarcinoma (ICC) by numerous evidences. However, as a well‐recognized suppressor of FGFR2 signalling, the clinical significance of Sprouty (SPRY) family of ICC has not been investigated. In our study, the expressions of SPRY1‐4 in 20 pairs of fresh tumour tissues were detected with qPCR, and in 108 cases of paraffin‐embedded tissues with immunohistochemistry. The prognostic value of SPRY family in ICC was estimated with univariate analysis and multivariate analysis. As a result, SPRY2 was identified as an independent prognostic biomarker predicting favourable prognosis of ICC. High SPRY2 expression was correlated with good differentiation of ICC. With silencing SPRY2 expression, we demonstrated that SPRY2 could suppress FGFR2‐induced ERK phosphorylation, migration, invasion and epithelial‐mesenchymal transition (EMT) under FGF1 stimulation. By overexpressing SPRY2‐wide type or SPRY2‐Y55F, the tyrosine‐55 of SPRY2 was demonstrated to be essential in suppressing ERK phosphorylation, tumour invasion and EMT of ICC cells. In conclusion, SPRY2 was correlated with favourable prognosis of ICC via suppressing FGFR2‐induced ERK phosphorylation, invasion and EMT. The phosphorylation of SPRY2‐Y55 was required in this tumour‐suppressing function of SPRY2.

## 1 INTRODUCTION

Cholangiocarcinoma is a type of malignancy originating from the epithelial cell of biliary tree. According to the arising locations, cholangiocarcinoma could be further classified into intrahepatic cholangiocarcinoma (ICC), perihilar cholangiocarcinoma and distant cholangiocarcinoma.^1^ ICC only accounts for 10% of all cholangiocarcinoma, but its morbidity is increasing rapidly. In recent years, there are significant breakthroughs in the molecular profile and classification of ICC,^2^ resulting in the identification of new genetic mutations and molecular biomarkers. Among all the defined biomarkers, FGFR2 is well acknowledged to promote the progression and lead to poor prognosis of ICC. Two independent studies reported the presence of FGFR2 fusion genes in ICC including FGFR2‐AHCYL1, FGFR2‐BICC1 and FGFR2‐CCDC6,^3^, ^4^, ^5^ which could activate mitogen‐activated protein kinases (MAPK), tumour growth and in vivo tumorigenesis via stimulating the kinase activity of FGFR2.^3^ With Integrated genomewide and whole transcriptome sequence analyses, another independent study also demonstrated that FGFR2 downstream signalling pathway was ectopically activated and could be a putative therapeutic target.^6^ The autocrine loop of FGFR activation is also demonstrated to promote cholangiocarcinoma progression.^7^ In our previous study, we also proved that FGFR4 overexpression correlated with poor prognosis of ICC via promoting proliferation and invasion.^8^

Fibroblast growth factor receptors are stimulated by binding with FGFs and function as serine/threonine kinases by activating downstream Ras‐MAPK‐ERK pathway. During this process, sprouty (SPRY) family, comprising of four isoforms (SPRY 1 ‐ 4), are the key feedback inhibitors of FGFR‐induced Ras‐MAPK‐ERK pathway.^9^ SPRY can inhibit ERK phosphorylation via modulating different levels of the FGFR‐RAS pathway.^10^ For example, SPRY2 evidently binds to growth factor receptor‐bound protein 2 (GRB2), thereby negatively regulating downstream signalling of FGFR.^11^ Deregulation or dysfunction of SPRY was proved to result in pathological conditions such as oncogenesis or cancer progression including breast cancer, hepatocellular carcioma and prostate cancer, etc.^12^, ^13^, ^14^, ^15^, ^16^, ^17^, ^18^ Recently, we demonstrated that SPRY2 was correlated with favourable prognosis of gastric adenocarcinoma via suppressing FGFR2‐induced ERK phosphorylation and cancer progression.^19^ Although SPRY family has a distinct feedback inhibitory effect on FGFR signalling, and FGFR2 was demonstrated to promote ICC progression in numerous previous studies, the expression and clinical significance of SPRY2 family in ICC has never been elucidated. Here we detected the expression of SPRY family in 108 paraffin‐embedded ICCs and 20 pairs of fresh ICC tissues, and further evaluated the clinical significance of SPRY2 by analyzing the correlation between SPRY expression, tumour progression and prognosis.

## 2 PATIENTS AND METHODS

The primary cohort consisted of 254 patients who underwent radical resection of ICC from 2002 to 2015 in Qilu Hospital of Shandong University. The test cohort consisting of 108 patients was selected according to criteria as follows: (a) survival time more than 3 months, (b) available clinical follow‐up data, (c) no history of chemotherapy or radiotherapy. The average age of test cohort was 56.5 years old, ranging from 28 to 83 years old. Moreover, twenty pairs of fresh ICC tissues and the corresponding adjacent tissues were collected prospectively. The tissues were preserved in liquid nitrogen immediately after surgical resection for quantitative PCR (qPCR) detection. All the specimens were obtained with prior consent of patients and the approval of Ethics Committee of Qilu Hospital affiliated to Shandong University. The pathologic tumour‐node‐metastasis (pTNM) staging was based on the 8th staging classification of AJCC/UICC(2017). The protocol of this study was managed according to the requirement of Reporting Recommendations for Tumor Marker Prognostic Studies (REMARK).^20^

### 2.1 Tissue microarrays and immunohistochemistry

The tissue microarrays (TMA) of formalin‐fixed and paraffin‐embedded tissue sections were made as to previous report.^21^ Before immunohistochemistry (IHC) detection, haematoxylin and eosin staining were performed to confirm the histological characterization of all samples. The protocol of IHC staining was described in detail before.^22^ The results of IHC were evaluated independently by two senior pathologists unaware of the clinical information. The IHC results were semi‐quantified by the IHC score, which was comprised of the score for staining intensity and the score for percentage of stained cells. The score for staining intensity was defined as negative (0), weak (1), moderate (2) and strong (3). The percentage of stained cells was scored as 1, <10% of cells were positive; 2, 10%‐50% of cells were positive; 3, >50% of cells were positive. The final IHC score was the product of the score for staining intensity multiplied by the score for percentage of stained cells, which ranged from 0 to 9. The score with the highest summary of sensitivity and specialty in ROC curve was set as cut‐off, which divided the cohort into low and high expression level. The cut‐offs of SPRY1,2,3,4 were 3.5, 4.5, 3.5 and 4.5, respectively.

### 2.2 Cell culture and reagents

The ICC cell lines RBE, HuCCT1 and HCCC9810 were all purchased from Cell Bank of the Chinese Academy of Sciences (Shanghai, China) and cultured in the RPMI‐1640 medium supplemented with 10% foetal bovine serum (Gibco, Waltham, MA) and 1% ampicillin/streptomycin (Gibco) in 5% CO_2_ resuscitation. Human recombinant FGF1 was purchased from PeproTech Company, and the inhibitor AP24534 was purchased from Selleck, the primary antibodies of SPRY1‐4, pan‐phospho‐Tyr, and β‐actin were bought from Santa Cruz Biotechnology (Santa Cruz, CA), antibodies of FGFR2, Grb2, pFRS2‐Tyr436, p‐ERK‐Tyr202/204 and the EMT antibody sampler kit were purchased from the Cell Signaling Technology (Danvers, MA), antibody of p‐FGFR2‐Tyr769 was from Biorbyt Company (Cambridge, UK).

### 2.3 RNA extraction and RT‐PCR

The total mRNA of fresh tissues were extracted by Trizol agent (Invitrogen, Waltham, MA, USA) and RNeasy protect mini kit (Qiagen, Hilden, Germany). Complementary DNA (cDNA) synthesis was performed with qPCR‐RT‐Kit of Toyobo Company (Osaka, Japan). Quantitative PCR was realized with SYBR Green Master Mix and StepOnePlus system (Applied Biosystem, Waltham, MA). The ΔΔCt method was used to calculate the relative expression with GAPDH (glyceraldehyde 3‐phosphate dehydrogenase) as an internal control. The primers were designed as follows: SPRY1, forward 5′‐ATGGATCCCCAAAATCAACA‐3′, reverse 5′‐CGAGGAGCAGGTCTT TTCAC‐3′; SPRY2, forward 5′‐CCCCTCTGTCCAGATCCATA‐3′, reverse 5′‐CCCAAA TCTTCCTTGCTCAG‐3′; SPRY4, forward 5′‐AGCCTGTATTGAGCGGTTTG‐3′ and reverse 5′‐GGTCAATGGGTAGGATGGTG‐3′.

### 2.4 Plasmid construction and transfection

SPRY2‐Y55F mutation was generated with a Quikchange mutagenesis kit (Stratagene, La Jolla, CA) as previously report and verified by DNA sequencing.^23^ The transfection of SPRY2 Y55F, SPRY2‐WT and the siRNAs were performed with Lipofectamine 2000 (Invitrogen) according to the manual. The siRNA of FGFR2 and SPRY2 were obtained from the Genephama Company (Shanghai, China). Results of knockdown and overexpression were detected by Western blotting 48 hours after transfection.

### 2.5 Western blotting and analysis

Cells were lysed by lysis buffer added with proteinase inhibitor cocktail (1% NP‐40, 10 mmol/L Tris‐HCl, pH 7.4, 150 mmol/L NaCl, 5 mmol/L EDTA, 1 mmol/L sodium vanadate, and 10 μg leupeptin, 1 μg aprotinin, 1 μg pepstatin, 1 μg antipain, and 30 μg phenylmethylsulfonyl fluoride per mL). The lysed cells were centrifuged at 10 000× g for 30 minutes and the precipitation was discarded. Cellular protein concentration was quantified with Bradford method (Tiangen Biotech, Beijing, China), and equal amount of 10 μg protein was used for SDS‐PAGE. After transferred to nitrocellulose membranes (PALL, Port Washington, NY) and incubated in primary antibody (1:1000) at 4°C overnight and then in HRP‐conjugated secondary antibody (Beyotime, Beijing, China), the proteins were finally visualized by incubation in ECL agent (Merck Millipore,Kenilworth, NJ).

### 2.6 Scratch wound healing assay

Scratch would healing assay was performed with CytoSelect™ 24‐well wound healing assay (Cell Biolabs, San Diego, CA) according to the manual. Cells were transfected with siRNA of plasmid 48 hours before the test. FGF1 at concentration of 10 ng/mL was added to stimulate FGFR signalling if needed. Percentage of wound healing = migrated cell surface area/total surface area × 100%.

### 2.7 Matrigel invasion assay

Tumour invasion was evaluated in 8‐μm‐pore Matrigel‐coated transwells (BD Biosciences, Franklin Lakes, NJ). Cells were passaged into the matrigel invasion chambers and cultured for 6 hours for adherence, and then incubated in 1%‐serum‐containing medium with 100 ng/mL FGF1 for 12 hours. After incubating for 24 hours, cells were fixed by 4% paraformaldehyde for 30 minutes and stained by 0.05% gentian violet for 1 hour. Cells on the upper surface were removed with a cotton swab and the cell number on the lower surface was counted from 10 random fields. Cell numbers of control group were set as a baseline and fold change was calculated by ratio to control group. Statistical significance was evaluated by Student's *t* test.

### 2.8 Statistical analysis

SPSS 22.0 software was used to perform all the statistical analyses without special illustration. The correlations between clinicopathological features and biomarkers were assessed by χ2 test. The survival curves were displayed with Kaplan‐Meier method, and the survival curve differences in different groups were analysed with log‐rank test. Cox proportional hazards regression model was applied to identify the independent prognostic factors. The statistical differences of different groups in would healing assay of transwell assay was evaluated with ANOVA test or Student's *t* test. *P*‐values <0.05 was considered to be statistically significant.

## 3 RESULTS

### 3.1 Expression of SPRY family in ICC

**Figure 1:**
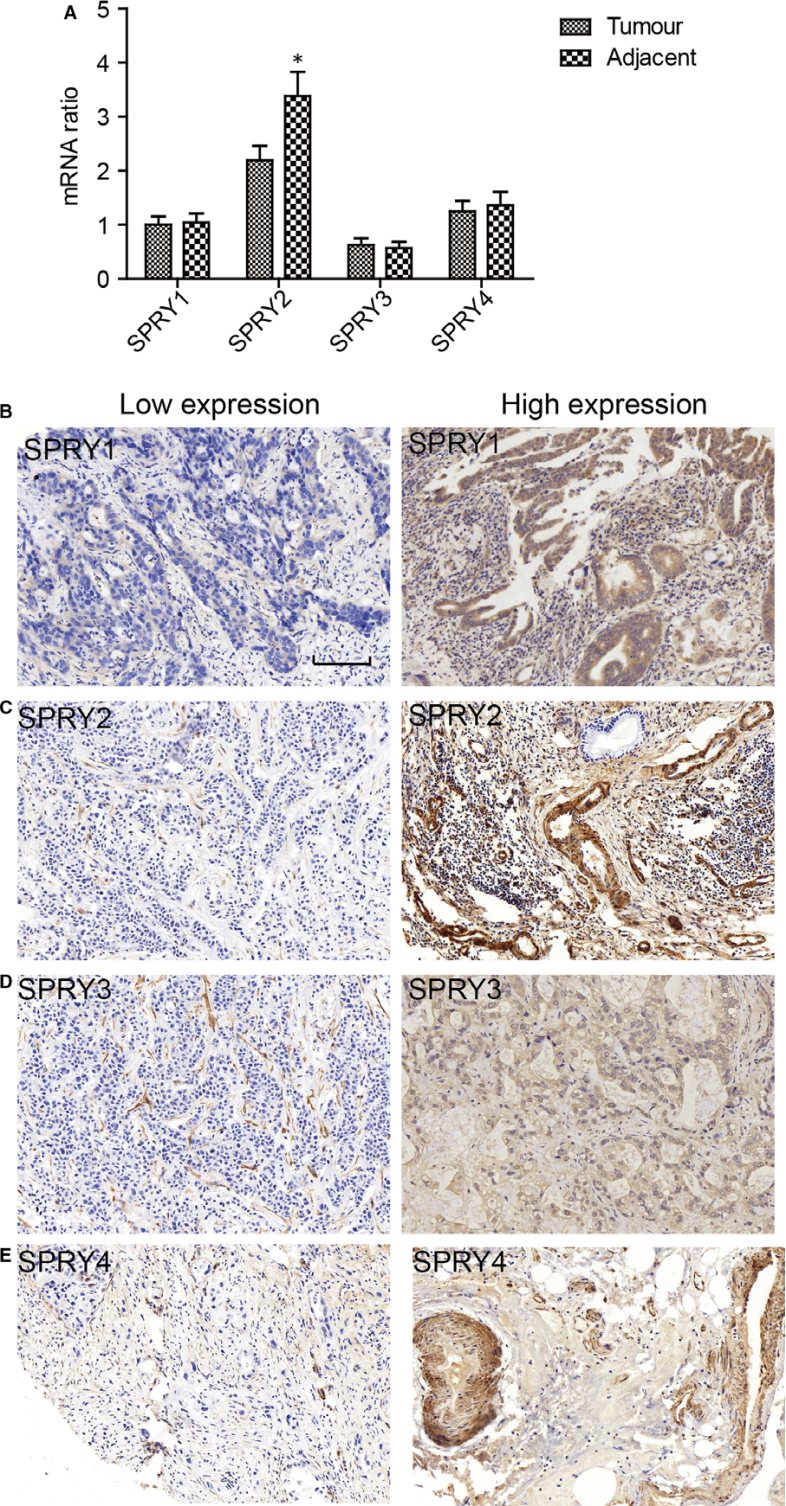
The expression of SPRY family in ICC. A, The mRNA level of SPRY1‐4 in 20 pairs of ICC tissues and adjacent tissues. B‐E, The representative images of low expression and high expression of SPRY1 (B), SPRY2 (C), SPRY3 (D) and SPRY4 (E). Scale bar:100 μm

The mRNA levels of SPRY family, including SPRY1‐4, were detected with RT‐PCR in 20 pairs of ICC tissues and adjacent tissues (Figure 1A). SPRY2 had relatively higher mRNA level compared with other SPRY members and its mRNA level in adjacent tissues was significantly higher than in tumour tissues, suggesting the potential tumour‐suppressing role of SPRY2 in ICC oncogenesis. The expressions of SPRY members were further investigated with IHC in 108 paraffin‐bedded specimens. According to the cut‐off determined by ROC curve, the cohort was divided into the high expression and low expression group (Figure 1B‐E). In ICC tissues, the expression of SPRY1‐4 was mostly observed in cytoplasm.

### 3.2 Prognostic value of SPRY family in ICC

**Figure 2:**
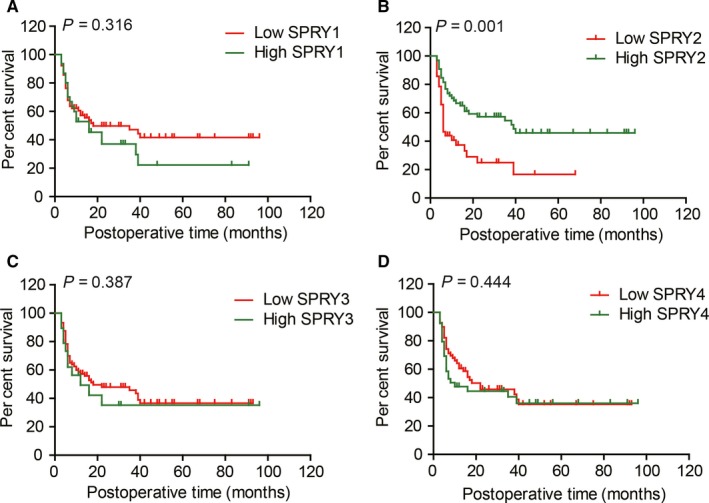
The correlation between SPRY family and the overall survival rates. A‐D, The survival curves of ICC patients were stratified with expression of SPRY1(A), SPRY2(B), SPRY3(C) and SPRY4(D). High expression of SPRY2 is significantly associated with low overall survival rates, while the other three SPRY members had no remarkable prognostic significance

The prognostic value of SPRY1‐4 was evaluated with both univariate analysis and multivariate analysis. Univariate analysis was first performed to screen the prognostic factors with Kaplan‐Meier method (Table 1). Among the clinicopathological factors, higher tumour size (*P *=* *0.027), positive lymph invasion (*P *<* *0.001) and metastasis (*P *=* *0.014), advanced TNM stage (*P *<* *0.001) could all predict the lower survival rates of ICC patients. In SPRY family, only SPRY2 (*P *=* *0.001) was confirmed as a prognostic biomarker indicating favourable prognosis in ICC (Figure 2A‐D).

**Table 1:** The prognostic significance of SPRY family and clinicopathological factors

Clinicopathologic parameters	3‐y survival%	*P* a	HR	95%CI	*P* b
Age (y)					
<60	40.7	0.974			
≥60	49.7				
Sex					
Male	40.6	0.656			
Female	47.7				
Tumour size (cm)					
<5 cm	58.6	0.027	1		
≥5 cm	35.6		1.53	0.81‐2.87	0.188
Differentiation					
Good	59.0	0.196			
Moderate	42.5				
Poor	35.5				
T stage					
T1 + T2	46.6	0.476			
T3 + T4	35.6				
N stage					
N0	55.5	<0.001	1		
N1	19.4		2.06	1.18‐3.61	0.011
M stage					
M0	45.8	0.014	1		
M1	0		1.42	0.48‐4.18	0.527
TNM stage					
I	70.8	<0.001			
II	35.8				
III	46.0				
IV	18.8				
Hepatolith					
No	43.3	0.993			
Yes	44.4				
SPRY1					
Low	47.2	0.316			
High	37.1				
SPRY2					
Low	16.6	0.001	1		
High	54.5		1.92	1.10‐3.34	0.021
SPRY3					
Low	46.5	0.387			
High	33.6				
SPRY4					
Low	45.8	0.444			
High	40.5				

HR, hazard ratio, CI, confidence internal.

a Calculated by log‐rank test.

b Calculated by Cox‐regression Hazard model.

**Table 2:** The correlation between SPRY2 and clinicopathological factors

Clinicopathologic parameters	n	Percentage (%)	SPRY2		
			Low	High	*P* a
Age (y)					
<60	67	62.04	25	42	0.689
≥60	41	37.96	17	24	
Sex					
Male	54	50.00	22	32	0.844
Female	54	50.00	20	34	
Tumour size (cm)					
<5	37	34.26	11	26	0.212
≥5	71	65.74	31	40	
Differentiation					
Good	21	19.44	8	13	0.036
Moderate	53	49.07	15	38	
Poor	34	31.48	19	15	
T stage					
T1 + T2	83	76.85	30	53	0.351
T3 + T4	25	23.15	12	13	
N stage					
N0	70	64.81	21	49	0.013
N1	38	35.19	21	17	
M stage					
M0	104	96.30	39	65	0.297
M1	4	3.70	3	1	
TNM stage					
I	37	33.33	10	27	0.014
II	17	15.74	6	11	
III	17	15.74	4	13	
IV	37	34.26	22	15	
Hepatolith					
No	93	86.11	37	56	0.778
Yes	15	13.89	5	10	
SPRY1					
Low	77	71.30	31	46	0.670
High	31	28.70	11	20	
SPRY3					
Low	86	79.63	31	55	0.327
High	22	20.37	11	11	
SPRY4					
Low	69	63.89	23	46	0.151
High	39	36.11	19	20	

a Calculated by Chi‐square test.

The prognostic factors in univariate analysis were all enrolled into Cox‐regression model for multivariate analysis (Table 2), except TNM stage because of its obvious interaction with T, N and M stage. In the multivariate analysis, N stage (*P *=* *0.011, HR* *=* *2.06) and SPRY2 expression (*P *=* *0.021, HR* *=* *1.92) were identified as independent prognostic factors of ICC.

### 3.3 Correlation between SPRY2 and clinicopathological factors

The correlation between SPRY2 and clinicopathological factors was analysed with Chi‐Square test to screen the potential tumour progression processes influenced by SPRY2 (Table 2). Low expression of SPRY2 was significantly correlated with poor differentiation (*P *=* *0.036) and positive lymphatic invasion (*P *=* *0.013), which suggested that SPRY2 may be involved in the process of ICC invasion. TNM stage was also associated with SPRY2 expression, and this may be the secondary consequence because of the correlation between SPRY2 and N stage.

### 3.4 SPRY2 expression is associated with differentiation of ICC

**Figure 3:**
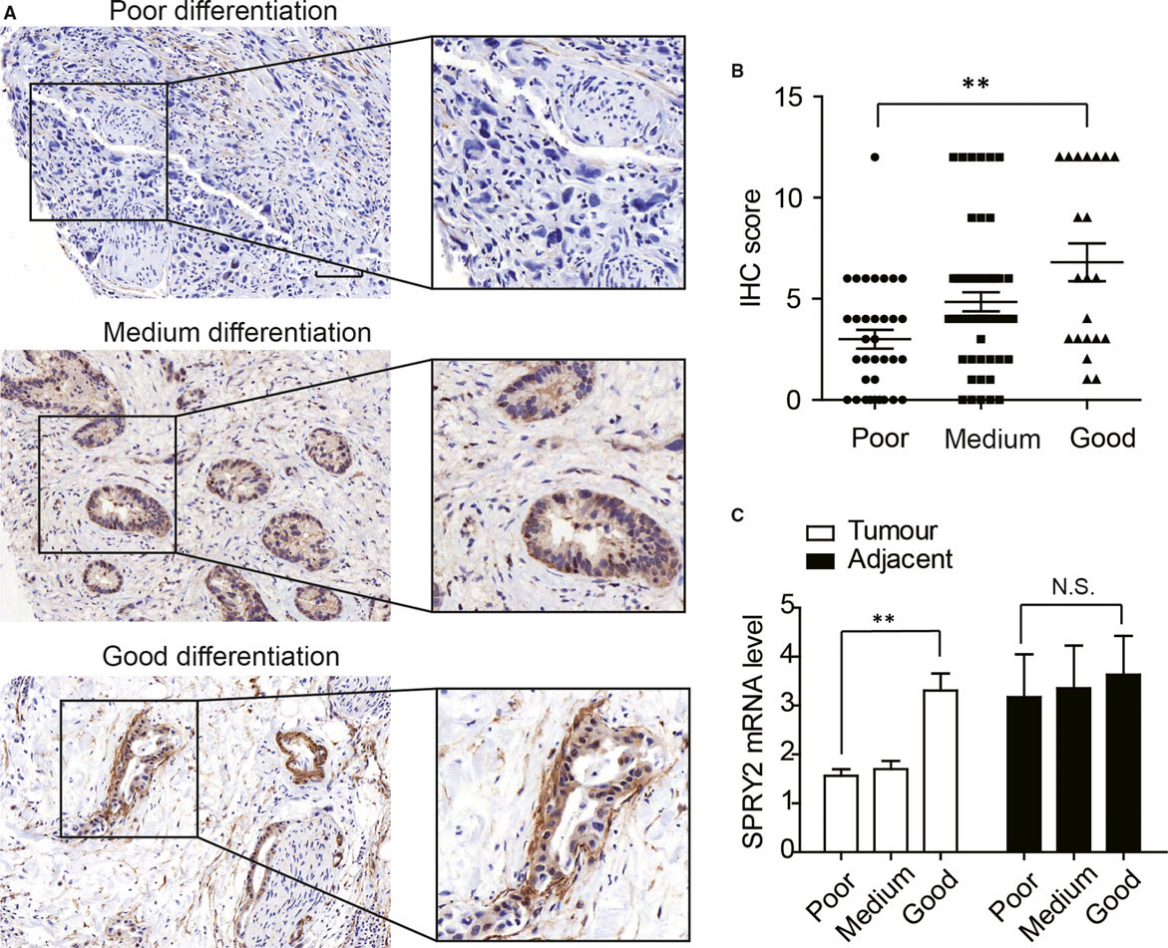
The correlation between SPRY2 and ICC differentiation. A, The representative images of ICC tissues with different differentiation and SPRY2 expression. ICCs with higher SPRY2 expression appeared to have better differentiation. B, The SPRY2 IHC score of ICC patients with different differentiation. Patients with good differentiation had higher expression of SPRY2 compared with those with poor differentiation. C, ICC with good differentiation had higher SPRY2 mRNA compared with those with poor differentiation, and the mRNA of SPRY2 in adjacent tissues had no significant difference. N.S. means not significant, **P < 0.01, ***P < 0.001

With Chi‐Square test, we demonstrated that poorly differentiated cases appeared to have lower SPRY2 expression (Figure 3A), so we further stratified the test cohort according to tumour differentiation and compared the SPRY2 expression. Consequently, ICC with good differentiation had significantly higher SPRY2 expression compared with ICC with poor differentiation (Figure 3B). Moreover, we compared the SPRY2 mRNA level of different differentiation in fresh ICC tissues. Patients with poorly differentiated ICC had lower mRNA level of SPRY2 compared with those with well‐differentiated ICC. However, the SPRY2 levels in adjacent tissues had no significant difference (Figure 3C). These results suggested that overexpression of SPRY2 could correlate to better differentiation of ICC.

### 3.5 SPRY2 suppresses FGFR2‐induced phosphorylation of ERK

**Figure 4:**
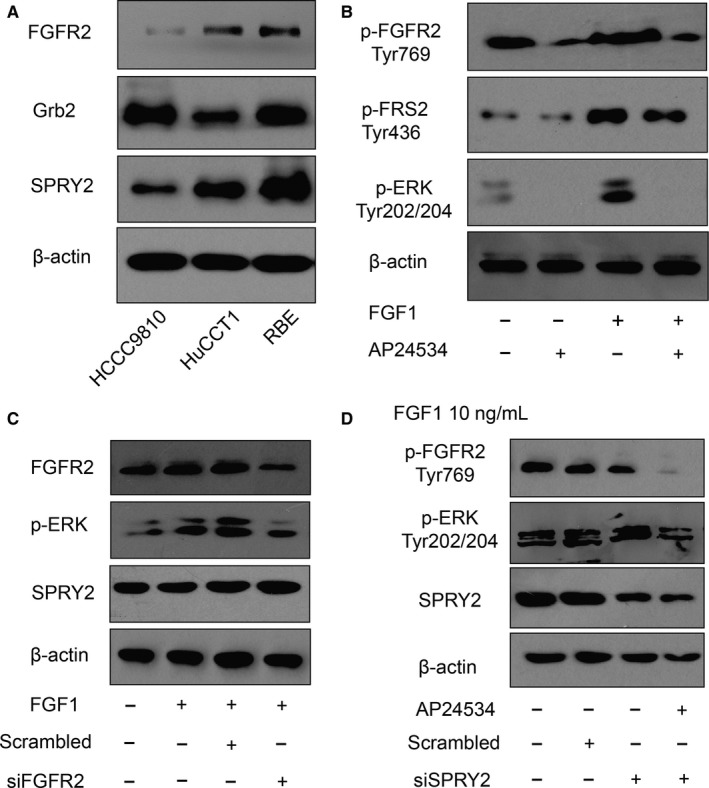
SPRY2 suppressed FGFR2‐induced ERK phosphorylation in ICC cells. A, The basal expression of FGFR2, Grb2 and SPRY2 in ICC cell lines HCCC9810, HUCCT1 and SPRY2. B, FGFR inhibitor AP24354 suppressed FGF1‐induced phosphorylation of FGFR2, FRS2 and ERK in RBE cells. RBE cells were stimulated with 10 ng/mL FGF1 for 5 min with/without 1 μmol/L AP24354 for 30 min. C, FGFR2 was required in FGF1‐induced ERK phosphorylation. The FGFR2 expression in RBE cells was silenced with transfection of FGFR2 siRNA 48 h before treatment with 10 ng/mL FGF1 for 5 min. FGFR2 knockdown significantly decreased ERK phosphorylation with FGF1 stimulation. D, SPRY2 and AP24354 could attenuate the FGFR2‐mediated ERK phosphorylation. RBE cells were transfected with SPRY2 siRNA or scrambled siRNA as control for SPRY2 knockdown. All cells were treated with 10 ng/mL FGF1 with/without 1 μmol/L AP24354. AP24354 impaired phosphorylation of FGFR2 and ERK, while SPRY2 knockdown amplified ERK phosphorylation

As SPRY2 is a recognized suppressor of ERK phosphorylation triggered by FGF‐FGFR signalling, we detected the function of SPRY2 in FGFR2‐induced phosphorylation of ERK of ICC cells. The intrinsic expressions of SPRY2 in ICC cell lines were detected, including HCCC9810, HuCCT1 and RBE cells. RBE cells had relatively higher SPRY2 and FGFR2 expression (Figure 4A). With the incubation of 10 ng/mL FGF1 and/or 1 μmol/L FGFR2 inhibitor AP24534, we demonstrated that FGFR2 phosphorylation could induce the phosphorylation of FRS2 and ERK of RBE cells (Figure 4B). With silencing FGFR2 or SPRY2 expression with siRNA, FGFR2 was demonstrated to be required in FGF1‐induced ERK phosphorylation in RBE cells (Figure 4C), and SPRY2 could suppress the FGFR2‐mediated ERK phosphorylation (Figure 4D).

### 3.6 SPRY2 suppresses EMT and invasion of ICC

**Figure 5:**
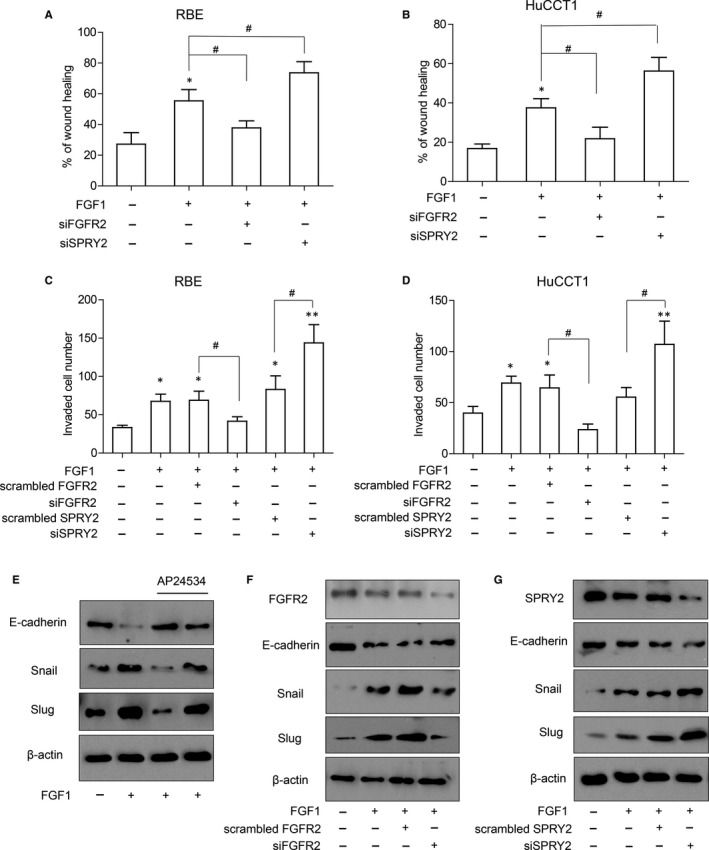
SPRY2 attenuated FGFR2‐induced invasion and EMT. A‐B, The migration of RBE (A) or HuCCT1 cells (B) were enhanced by silencing SPRY2 and impaired by silencing FGFR2. 10 ng/mL FGF1 stimulation was used to incubate RBE or HuCCT1 cells for 12 h after transfection of FGFR2 or SPRY2 siRNA. Would healing assay was performed to evaluate the migration ability of RBE or HuCCT1 cells after silencing FGFR2 or SPRY2. *P <* *0.05 compared cells without FGF1 activation, ^#^ *P *<* *0.05. C‐D, With 10 ng/mL FGF1 stimulation, SPRY2 attenuated FGFR2‐induced invasion in RBE (C) or HuCCT1 cells (D). RBE or HuCCT1 cells were transfected with scrambled siRNA, FGFR2 siRNA or SPRY2 siRNA, respectively. Forty‐eight hours after transfection, the invasion of cells was detected with transwell assay. *P <* *0.05, **P <* *0.01 compared with cells without FGF1 activation, ^#^ *P *<* *0.05. E, FGF1 could enhance EMT via FGFR‐mediated pathway. RBE cells were incubated with 10 ng/mL FGF1 or 1 μmol/L AP24354 for 24 h and EMT markers including E‐cadherin, Snail, Slug were detected with Western blotting. F, FGFR2 was required in FGF1‐induced EMT. RBE cells were transfected with scrambled siRNA or FGFR2 siRNA 24 h before treated with 10 ng/mL FGF1 for 24 h. EMT markers including E‐cadherin, Snail, Slug after silencing FGFR2 were detected with Western blotting. G, SPRY2 could suppress FGF1‐induced EMT. After knocking down SPRY2 expression with SPRY2 siRNA and stimulating with 10 ng/mL FGF1, EMT markers were detected with Western blotting

In the clinical analyzation of Table 2, we observed that SPRY2 expression was significantly associated with ICC differentiation. As tumour differentiation is an important factor determining tumour invasion, we investigated the role of SPRY2 on ICC cell migration and invasion. With wound healing assay, we proved that FGFR2 knockdown notably impaired the tumour cell migration while silencing SPRY2 could promote migration of RBE or HuCCT‐1 cells (Figure 5A,B). Similar results were observed in transwell assay for invasion detection. FGF1 stimulation could remarkably increase tumour invasion with normal FGFR2 expression, but the invasion of RBE or HuCCT‐1 was decreased when FGFR2 was knocked down and increased when SPRY2 was knocked down (Figure 5C,D). The above results indicated the role of SPRY2 as a tumour suppressor to ICC migration and invasion.

EMT is a generally accepted process inducing invasion and migration and SPRY2 has been reported to influence EMT process in ovarian cancer^24^; therefore, the influence of SPRY2 on EMT process of ICC was investigated. FGFR inhibitor AP24534 could suppress FGF1‐induced expression of Slug and Snail, and rescue the decrease in E‐cadherin, indicating AP24534 could attenuate the FGF1‐induced EMT process of ICC (Figure 5E). With knocking down FGFR2 or SPRY2, we demonstrated that FGFR2 was necessary for the FGF1‐induced EMT and SPRY2 could repress the FGFR2‐mediated EMT process (Figure 5F,G).

### 3.7 Phosphorylation of tyrosine 55 is essential for SPRY2 suppressing ERK phosphorylation and EMT

**Figure 6:**
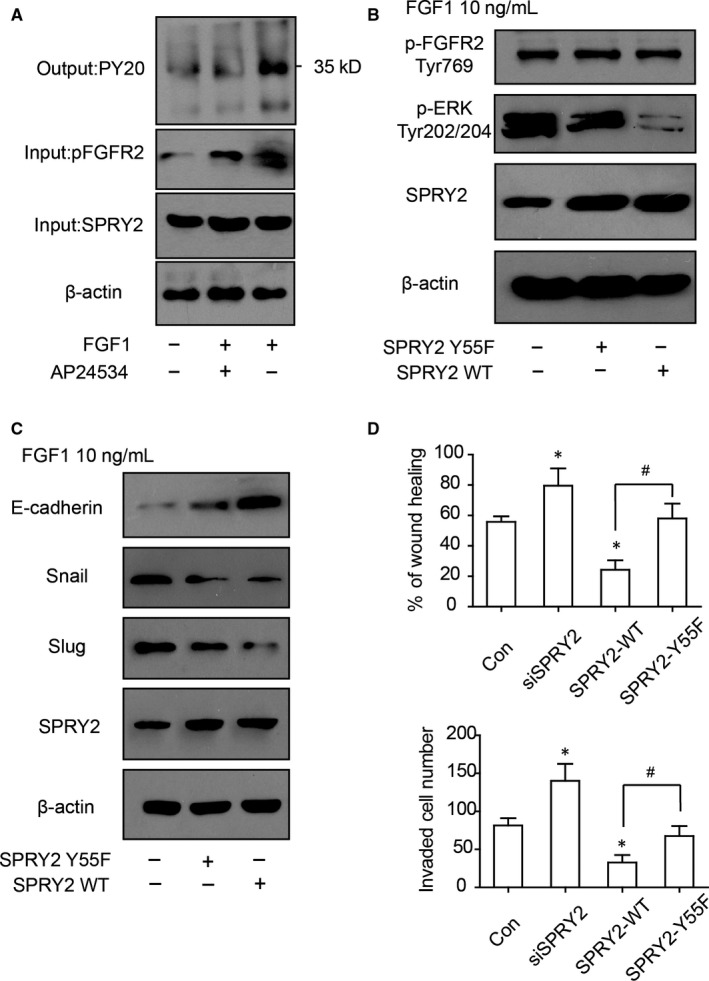
Y55 phosphorylation of SPRY2 was essential for repressing FGF1‐induced ERK phosphorylation and EMT. A, The phosphorylation of SPRY2 increased with FGF1 stimulation. RBE cells were treated with 10 ng/mL FGF1 and/or 1 μmol/L AP24354 for 30 min and lysed. SPRY2 antibody binding with Protein A/G was used to immunoprecipitate SPRY2 and common phosphorylation antibody pY20 was applied to detect the phosphorylation state of SPRY2. B, SPRY2‐Y55 was essential for antagonizing ERK phosphorylation. RBE cells were transfected with plasmid carrying wild‐type SPRY2 or SPRY2‐Y55F 24 h before 10 ng/mL FGF1 stimulation. ERK phosphorylation of cells transfected with SPRY2‐WT was significantly lower than SPRY2‐Y55F. C, SPRY2‐Y55 was required for suppression of EMT by SPRY2. With FGF1 stimulation, RBE cells transfected with wild‐type SPRY2 had lower E‐cadherin, higher Snail and Slug expression compared with cells transfected with SPRY2‐Y55F. D‐E, SPRY2‐Y55 was required for SPRY2‐repressing migration and invasion of ICC cells. 24 h after transfected with wild‐type SPRY2 or SPRY2‐Y55F, RBE cells were stimulated with FGF1 for 12 h. Cell migration and invasion was evaluated with would healing assay (D) and transwell assay (E), respectively. *P <* *0.05 compared with control group, ^#^ *P *<* *0.05

According to previous studies, The tyrosine55 of SPRY2 is considered to be responsible for binding with Grb2 and recognizing FGF‐specific ERK‐activating pathway,^25^ so the function of SPRY2‐Tyr55 in RBE cells was further investigated. SPRY2 was immunoprecipitated via protein A/G and its phosphorylating status was detected with pan‐phospho‐Tyr antibody. The phosphorylation of SPRY2 was increased after FGF1 stimulation and decreased with AP24534 incubation (Figure 6A), suggesting that SPRY2 phosphorylation was correlated with FGF1 activation. To verify the function of SPRY2 Tyr55, the plasmids carrying wild‐type SPRY2 (SPRY2‐WT) or mutated SPRY2 (SPRY2‐Y55F) were transfected into RBE cells. ERK phosphorylation of cells transfected with SPRY2‐Y55F was remarkably higher than those transfected with SPRY2‐WT (Figure 6B), indicating Tyr‐55 was essential for dephosphorylating ERK by SPRY2. Moreover, the mutation of SPRY2 Tyr‐55 also impaired its function of suppressing EMT (Figure 6C). With wound healing assay and transwell assay, cells with SPRY2‐Y55F overexpression had significantly more aggressive migration and invasion than cells overexpressing SPRY2‐WT(Figure 6D,E), indicating Tyr‐55 was required in ICC migration and invasion.

## 4 DISCUSSION

The breakthroughs in precise classification by molecular profile and the definition of new biomarkers are the basis of new targeted therapy and treating strategy. FGFR2 is a well‐acknowledged prognostic biomarker in ICC, and ICC with FGFR2 fusion genes is considered as a unique molecular subtype of ICC.^3^ Many FGFR inhibitors are demonstrated to be effective to suppress ICC progression in xenograft mouse models,^5^ and several are applied in clinical trial of different phase.^26^ The agents targeting FGFR are nowadays the most promising target therapy in ICC treatment. Almost all the attempts aim to inhibit activation of FGFR2, and our findings of that SPRY2 antagonizes FGFR2 signalling in ICC could bring a new insight to the emerging therapy besides the FGFR2 inhibitor. Drugs activating SPRY2 or inhibiting the downstream FGFR2 signalling could also play similar effects on suppressing ICC progression, like inhibiting FGFR2 directly with FGFR2 inhibitor. In our study, high expression of SPRY2 was demonstrated to indicate favourable prognosis via antagonizing FGF‐FGFR2 signalling. This is the first study on the tumour‐suppressing function of SPRY2 in ICC as far as we know. Moreover, SPRY2 was significantly correlated with tumour differentiation and lymphatic invasion, instead of other factors like tumour size, suggesting that SPRY2 may be involved in the tumour invasion process like EMT or cell differentiation rather than proliferation. In fact, SPRY2 could antagonize the signalling initiated by many kinds of receptor tyrosine kinase, like EGFR, IGFR or FGFR, with different mechanisms. Even specific to FGFR signalling, the intact theory or how SPRY antagonizes FGFR‐mediated ERK phosphorylation is still in controversy. There are several explanations of how SPRY inhibits the Ras/ERK pathway in different levels. SPRY and SPRED (SPRY‐related EVH1 domain‐containing protein) can inhibit Raf activity by binding with it via a conserved sequence in the C terminus.^27^ SPRY2 could also interact with SH2 domain of Grb2 and attenuate ERK phosphorylation.^25^ Due to the redundancy of SPRY family and their multitask mechanism to suppress ERK phosphorylation, the SPRY is generally considered to be cell‐ and context‐specific. Our finding that only SPRY2 in SPRY family correlates to favourable prognosis also supports this suggestion. It is not the main focus to dig out the binding protein of SPRY2 was not identified and the underlying molecular mechanism in ICC, but it is certainly an interesting topic worthy of further study.

In conclusion, we investigated the expression of SPRY family in ICC and identified SPRY2 as an independent prognostic biomarker of ICC. With experiments in vitro, we demonstrated that SPRY2 could inhibit FGFR2‐induced ERK phosphorylation, EMT, cell migration and invasion, which required the phosphorylation of SPRY2‐Tyr55. Our findings could expand the understanding of FGFR signalling in ICC and provide new insights into ICC targeted therapy like developing drugs to antagonize downstream molecules of FGFR signalling.

## CONFLICT OF INTEREST

None.
