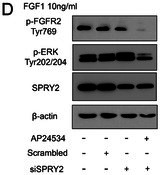# Correction to “Sprouty2 Suppresses Progression and Correlates to Favourable Prognosis of Intrahepatic Cholangiocarcinoma via Antagonizing FGFR2 Signalling”

**DOI:** 10.1111/jcmm.71149

**Published:** 2026-04-22

Yun‐Fei Xu, Hong‐Da Liu, Zeng‐Li Liu, et al., “Sprouty2 Suppresses Progression and Correlates to Favorable Prognosis of Intrahepatic Cholangiocarcinoma via Antagonizing FGFR2 Signaling,” *Journal of Cellular and Molecular Medicine* 22, no. 11 (2018): 5596–5606, https://doi.org/10.1111/jcmm.13833.

In this article, the images of p‐ERK and SPRY2 in Figure 4D were misused during manuscript preparation. The correct Figure 4D is shown below. Moreover, the labeling of Figure 5E should be FGF “− + − +” instead of “− + + +”.

The authors sincerely apologize for this error, and confirm all results and conclusions of this article remain unchanged.